# Comparison of conventional internal limiting membrane versus pars plana vitrectomy without peeling for small idiopathic macular hole

**DOI:** 10.1186/s40942-024-00599-5

**Published:** 2024-10-24

**Authors:** Maria Ludovica Ruggeri, Alberto Quarta, Paola Marolo, Lucio Zeppa, Lorenzo Motta, Matteo Gironi, Lisa Toto, Michele Reibaldi, Rodolfo Mastropasqua

**Affiliations:** 1 Ophthalmology Clinic, Department of Medicine and Science of Ageing, University G. D’Annunzio Chieti-Pescara, via dei Vestini 31, Chieti, 66100 Italy; 2 Department of Surgical Sciences Eye Clinic Section, University of Turin, Turin, 10122 Italy; 3 AORN Moscati Avellino, Avellino, Italy; 4 Ophthalmology Unit, Department of Neuroscience, University of Padova, Padova, 35128 Italy; 5 Department of Neurosciences, Imaging and Clinical Sciences, University “G. d’Annunzio” Chieti-Pescara, Chieti, Italy

**Keywords:** FTMHs, ILM peeling, OCT, Vitreoretinal surgery

## Abstract

**Background:**

The aim of this study was to compare functional and anatomical changes in patients with small full thickness macular holes (FTMHs) who underwent pars plana vitrectomy (PPV) with or without Internal limiting membrane (ILM) peeling.

**Methods:**

42 eyes of 42 patients diagnosed for FTMHs (< 250 micron) were included in our prospective interventional study. Main outcome measures were: Best Corrected Visual Acuity (BCVA), Macular hole closure rate, Ellipsoid Zone (EZ) and External Limiting Membrane (ELM) recover, Vessel Density in both Superficial (VDSCP) and deep (VDDCP) capillary plexus, Macular pigment Optical density (MPOD) and mean Central Macular Sensitivity (CMS).Patients were randomly divided into “peeling group” (21 patients), in which the ILM peeling maneuver was performed and “no-peeling group” (21 patients) in which the ILM was not peeled off. Examinations were repeated one month (T1), three months (T2) and six months (T3) after surgery.

**Results:**

Although significant improvements in terms of MPOD, CMS, VDSCP and VDDCP over time (*p* < 0.001) no significant differences were found between the peeling and no peeling group. Conversely, FTMHs closure was achieved in all cases (100%) in the peeling group, whereas 10% of cases in the no peeling group experienced the hole re-opening at T3, with reported different rates of ELM/EZ recover between the two groups. Nevertheless, BCVA improved significantly (*p* < 0.001) but without significant differences between the two groups.

**Conclusions:**

No significant differences were found in terms of anatomical and functional outcomes between the peeling or not the ILM in small FTMHs at 6 months follow-up.

## Background

Full thickness macular hole (FTMH) is a common condition affecting 3.3 per 1000 people characterized by the presence of a full thickness foveal defect extending from the internal limiting membrane (ILM) to the photoreceptor layer [1, 2]. As a result, patients experience decreased visual acuity with central visual loss and metamorphopsia [3]. The pathogenetic cause of FTMH is mainly found in an anomalous posterior vitreous detachment (PVD), with the presence of pathological anteroposterior tractional forces between vitreous cortex and foveal and perifoveal areas as key factor for the disease development. Besides, recent studies have demonstrated that tangential tractional forces may enlarge the hole, by pulling away the foveal edges and damaging the foveal muller cells [4, 5]. A moderate percentage of macular traction causing FTMHs resolves spontaneously (10–32%), however, not always being accompanied by hole closure [6–8]. Uwaydat et al. in their retrospective collaborative study suggested an observation period for non-surgical hole closure in case of trauma, cystoid macular edema treatment and hole size < 200 μm [9]. Yuzawa et al. reported 6% of cases of spontaneous FTMH resolution in their study, despite the lack of identified factors involved in the process [10]. Although the epidemiology of FTMHs is well assessed, there is no clear established correlation between MH stage and vitreomacular traction (VMT). However, Optical Coherence Tomography (OCT) has permitted the study of the evolution from vitreomacular adhesion (VMA) to FTMH. The OCT-based classification proposed by the International Vitreomacular Traction Study (IVTS) group in 2013, has described the VMA as focal ( < = 1500 μm) or broad (> 1500 μm), and FTMHs into small (< 250 μm), medium (251–400 μm) and large (> 400 μm) basing on the hole narrowest horizontal linear width measured on OCT macular scans [11]. In the present classification, the status of vitreous is only considered as presence or absence of vitreous attachment. Pars Plana Vitrectomy (PPV) with gas or air tamponade has been considered as standard treatment for macular holes for years, by demonstrating to be effective in visual gain and hole closure [12–15]. Some studies have reported that additional ILM peeling may improve the surgery successful rate, defined as the hole closure after the first surgery from approximately 80% without ILM peeling to 96% with ILM peeling and has therefore become part of treatment [16–20]. However, it is uncertain whether in small holes it is always required. Over the years some concerns have been raised about possible negative effects of ILM peeling. Teresaki et al. studied the electrophysiologic changes occurring after ILM peeling in eyes with FTMH finding delay in implicit time and a reduction in the amplitude of the focal electroretinogram (ERG) b-wave in the ILM peeling group thus suggesting an incomplete Muller cells recovery [21]. Haritoglou et al. in their prospective study found the presence of macular changes in patients who underwent PPV with ILM peeling [22]. It is undoubtedly known that Muller cells provide architectural support to the retina and have a key role in retinal metabolic processes making ILM peeling a matter of debate for years. Thus, the aim of our prospective study was to compare functional and anatomical changes in patients with small FTMHs who underwent PPV with ILM peeling and PPV without ILM peeling before and after surgery.

## Methods

### Participants

In this prospective interventional study, 44 eyes of 44 patients (21 males and 23 females) with a definite diagnosis of small FTMHs were recruited at the Ophthalmology Clinic of University G. d’Annunzio, Chieti-Pescara, Italy. Patients were enrolled sequentially among those accessing to our Ophthalmology Clinic between May 2022 and January 2023. Small FTMH were defined according to the IVTS classification as focal defect extending from the ILM to the photoreceptor layer with the narrowest linear width at the OCT macular scan inferior to 250 μm [11]. Exclusion Criteria were: presence of PVD at presentation, secondary FTMHs, ocular surgery in the previous 6 months, elevated myopia (> 5 D), other diseases affecting the eye, ocular media opacities (according to the Lens Classification System III). The presence of PVD was considered an exclusion criteria since could represent a bias due to the unclear duration of PVD and therefore of the vitreoretinal interface status. This study adhered to the tenets of the Declaration of Helsinki and was approved by the institutional board of the University “G.d’Annunzio” of Chieti-Pescara, Department of Neuroscience, Imaging and Clinical Science (DNISC3-23). Patients provided informed consent for the study.

### Examinations

All patients underwent complete ophthalmic examination including BCVA evaluation (measured with the Logarithm of the minimum angle of resolution, LogMAR chart), Amsler examination, Goldmann applanation tonometry, axial length measurement (IOL Master Biometry, Carl Zeiss Meditec Jena, Germany) slit- lamp biomicroscopy, indirect fundus ophthalmoscopy.

**Fig. 1:**
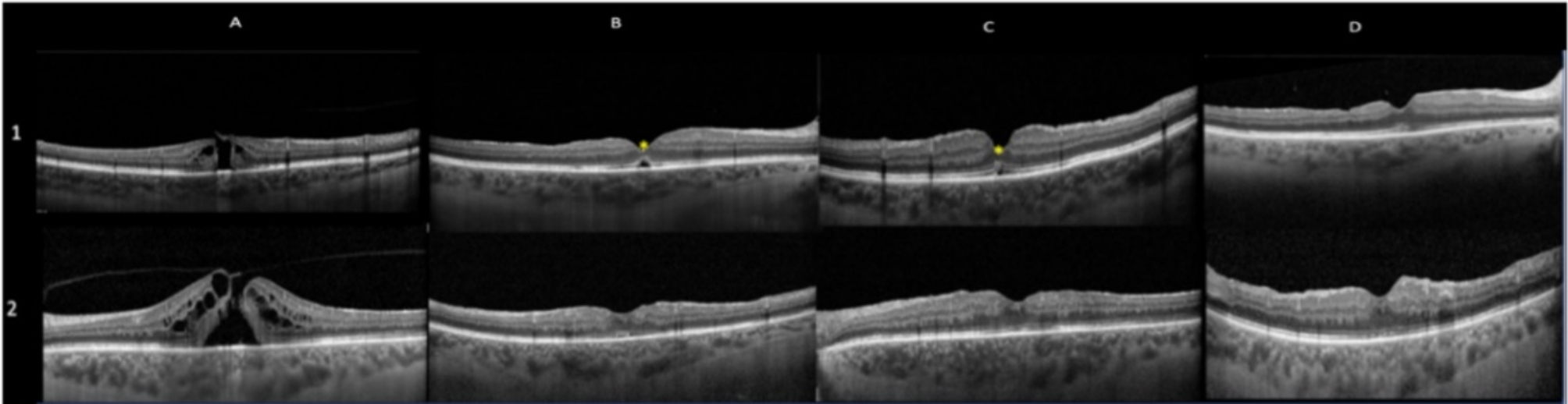
OCT linear macular scan at T0 (**A**), T1(**B**), T2 (**C**) and T3 (**D**) in both no peeling group (1) and peeling group (2). Case (1) shows a patient where incomplete EZ/ELM recover was noted during follow-up (*)

**Fig. 2:**
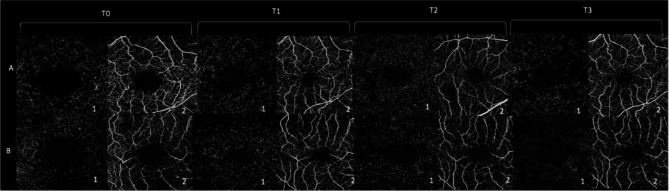
OCTA images of a peeling (**A**) and no peeling (**B**) patient of the deep (1) and superficial (2) capillary plexuses at different follow-ups times

**Fig. 3:**
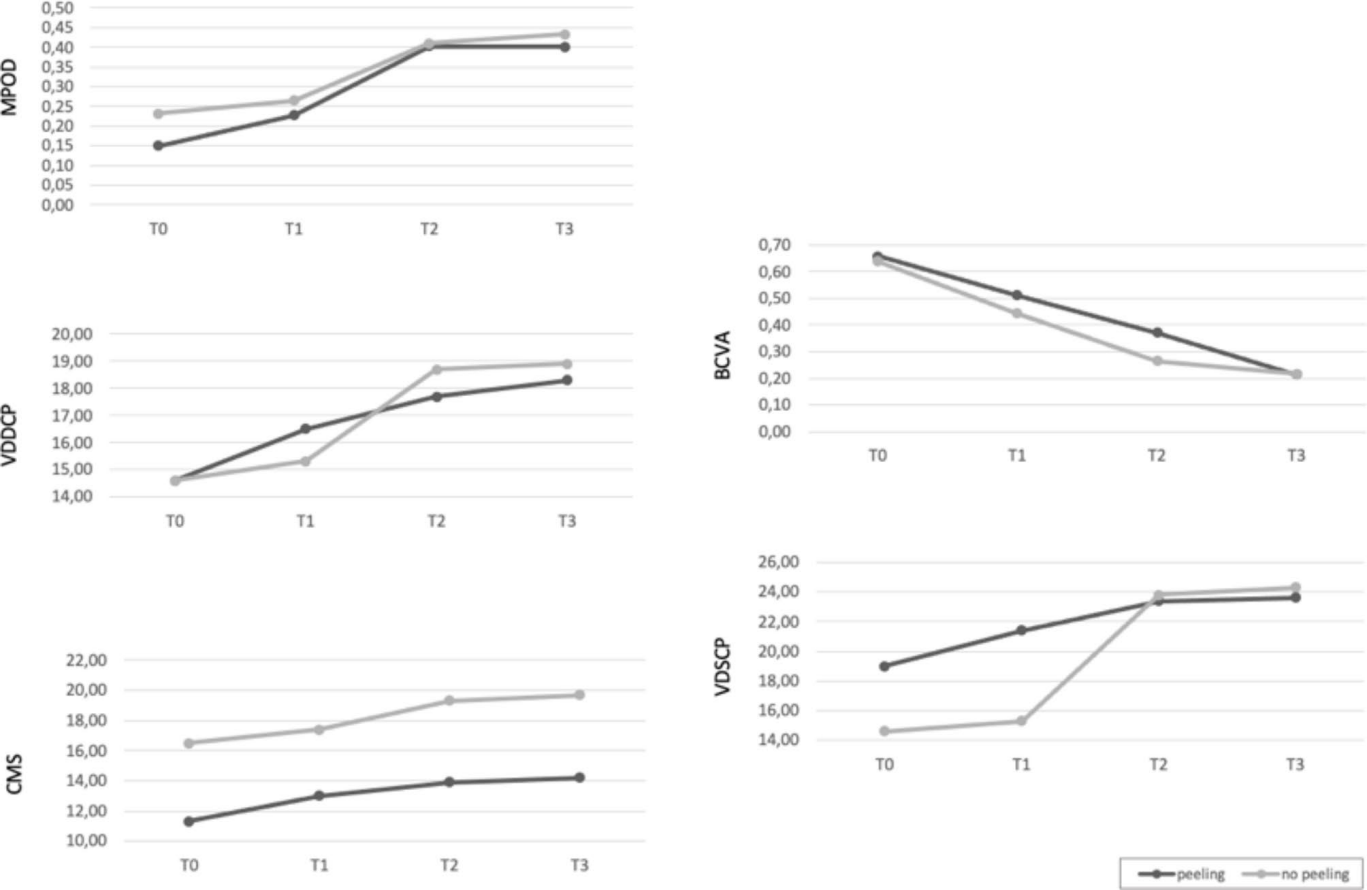
Baseline (T0), one month follow-up (T1), three months follow-up (T2) and six months follow-up (T3) mean values of MPOD, BCVA, VDSCP, VDDCP and CMS

In addition, spectral domain OCT (SD-OCT) was performed in all cases using Spectralis ^®^ HRA + OCT (Heidelberg Engineering; Heidelberg, Germany, Software Heyex). OCT Angiography (OCTA) was performed in all patients using the Plex-Elite 9000 device (Carl Zeiss AC, Jena, Germany). All patients were examined for Macular pigment Optical density (MPOD) using the Macular Pigment Screener (MPS) II (Elektron Technology, Cambridge, UK). More, mean central macular sensitivity (CMS) was evaluated with Microperimetry using Nidek-MP3 (NAVIS-ex 1,9.0 software, Nidek Technologies, Albignasego, Italy). All patients were candidate for surgery. Once diagnosed for macular hole, patients were scheduled for surgery at 6 weeks. On that day, OCT macular scan was performed prior to surgery and in case of occurrence of PVD or evidence of spontaneous hole closure, surgery was cancelled. Two patients were excluded by the study, due to the occurrence of spontaneous FTMH closure, thus, 42 eyes of 42 patients were analyzed. Patients were divided into two different groups (peeling group or no peeling group) by computer-based randomization program in a double- blinded fashion. The “peeling group” (twenty-one patients) received PPV with ILM peeling, The “no-peeling group” (twenty-one patients) was treated with PPV without ILM peeling. Examinations were performed at baseline (T0), one month (T1), three months (T2) and six months (T3) after surgery. (Figures 1, 2 and 3) Main outcomes measures were: BCVA, hole closure, Ellipsoid zone (EZ) and External Limiting Membrane (ELM) integrity, changes in Vessel density in both superficial (VDSCP) and deep plexuses (VDDCP), variation in mean CMS and MPOD changes over time.

### Optical coherence tomography analysis

SD-OCT was performed in all cases. The comprehensive acquisition protocol entailed the 49 horizontal raster dense linear B-scan and both linear horizontal and vertical B scan centered on the fovea. In addition, the Enhanced Depth Imaging (EDI) mode horizontal and vertical B-scans centered on the fovea and the 6-line radial and 25-line raster scans were performed in all enrolled patients [23]. Acquisitions following baseline were performed using the follow-up function. At baseline, the hole narrowest point was defined as the horizontal distance between the two edges of the hole at their narrowest length. Both measurements were performed using the inbuilt manual caliper on the horizontal line in the radial scan centered on the fovea [24]. At follow-ups, both hole closure and EZ and ELM reconstitution were evaluated on the same scan. All images were analyzed by two independent experienced readers. Images with poor signal strength (< 25) were excluded and thus repeated [25].

### Optical coherence tomography angiography analysis

Vessel density (VD) in both superficial and deep plexuses was evaluated at baseline, one month, three months and six months with PLEX Elite 9000 device (Carl Zeiss Meditec Inc., Dublin, CA, USA). Images were obtained using the FastTrack motion correction software. Low quality images (either due to motion artifact, incorrect segmentation, poor signal strength < 8) were excluded and thus repeated until good quality scans (with signal strength ≥ 8) were obtained [26–29].

(For each eye, a 3 × 3 mm, 6 × 6 mm and 12 × 12 mm volume scan centered on the fovea was performed. All vascular retinal layers were identified and segmented in the Superficial capillary plexus (SCP) and Deep Capillary Plexus (DCP) [30]. Segmentation was performed using the inbuilt algorithm. The projection resolved algorithm, which retains flow signals from blood vessels and suppresses projected flow signals in deep layers, was used [31]. Two different experienced retina specialists analyzed all images for segmentation accuracy, in case of disagreement, a third experienced retina specialist was consulted. Images were then exported as JPEG files and analyzed with ImageJ software (National Institutes of Health, Bethesda, MD, USA; available at http://rsb.info.nih.gov/jj/index.html). VD was defined as the percentage of the area occupied by vessels in a circular region of interest (ROI) and was calculated in the macular area (4 mm) in diameter located in the center of the foveal avascular zone) in the SCP and DCP through semiautomatic threshold segmentation as previously described [32].

### Macular pigment optical density analysis

The MPOD was assessed with the MPS II device (Elektron Technology, Cambridge, UK) by using a heterochromatic flicker photometry (HFP) technique. This technique exploits differences in term of intensity of blue and green wavelength flicker light, being the blue wavelength light (465 nm) absorbed by the macular pigment when compared to the green wavelength light (530 nm). Due to the higher concentration of macular pigment in the foveal area when compared to the para-fovea, this difference is measured and used to estimate the level of macular pigment that blocks the blue wavelength of light and arises the level to recognize blue light by photoreceptor cells. The density of macular pigment is derived by the subtraction of the para-foveal threshold from the foveal threshold. The main advantage of this technology is to exclude the interference of intermediate opacities such as cataracts [33]. All measurements were performed before pupil dilatation, and poor-quality registrations (defined as rejected values) were repeated until good measurements were obtained. When the latter was not possible, the result was recorded as non-measurable.

### Microperimetry analysis

Microperimetry was performed using the Nidek-MP3 (NAVIS-ex 1,9.0 software, Nidek Technologies, Albignasego, Italy). Patients were prepared 20 min before examination by receiving one drop of 1% tropicamide and 2.5% phenylephrine. The fellow eye was covered by a patch. The setting used was a red circle at 1.0˚ fixation target; monochromatic white background at 31.4 asb, dynamic range 34 db, Goldman I stimulus size with 200ms projection time and 4-to-2 threshold strategy. Automated eye tracking system integrated with the MP3 was used to fix eye movements. The color image was then acquired and overlapped to the retinal sensitivity point. Once assessed the fixation stability, the mean CMS was then analyzed.

### Surgical procedure

All surgeries were performed by the same experienced vitreo-retinal surgeon (RM) using the Constellation System (Alcon, Fort Worth, TX, USA). Patients were divided in two different groups (21 patients in the “Peeling group” and 21 patients in the “No Peeling group”). Phacoemulsification and intraocular lens (IOL) implantation were performed at the same time in 20 of 42 enrolled eyes, 12/20 (60%) patients were in the peeling group whereas 8/20 (40%) were in the “no-peeling” group. For all enrolled patients, a standard 25 gauge PPV was performed under local anesthesia. After the core vitrectomy, PVD was induced and the presence of vitreoschisis was checked with triamcinolone acetonide (Vitreal S, Fidia farmaceutici, Italy) .At this point of surgery, in the “Peeling group” ILM was stained with Membrane-dual blue (D.O.R.C., Netherlands) for 1 min, and after visualization, it was peeled off in a circular shape centered on the macular hole, with a range of 2–3 discs diameters in diameter. Besides, the ILM peeling maneuver was not performed in the “No peeling group”. Control of the retinal periphery with external scleral indentation was done in both groups. Fluid–air exchange was then performed. Vitreous cavity was filled with SF6 20% tamponade in all cases. All patients were invited to maintain a strict facedown position for three days postoperatively.

### Statistical analysis

Statistical evaluations were conducted employing the Jamovi project (2022). jamovi. (Version 2.3) [Computer Software]. Retrieved from https://www.jamovi.org. All tests were two-tailed, with a predetermined significance threshold of *p* < 0.05. Continuous variables were represented as mean ± standard deviation (SD), while categorical variables were depicted as mean values and/or proportions. A general linear model methodology was utilized to examine the interaction effect between the “between factor” and “within factor” (between factor: peeling versus no-peeling treatment; within factor, treatment time: baseline / pre-treatment / T0 vs. at the end of the 1st month of treatment / T1 vs. at the end of the 3rd month of treatment / T2 vs. at the end of the 6th month of treatment/T3). The sphericity of the covariance matrix was assessed through Mauchly’s test of sphericity; should the sphericity assumption be violated, the Greenhouse-Geisser epsilon (ε) correction was applied. To rigorously control type I error, post-hoc pairwise comparison tests were conducted using Holm’s method for multiple comparisons. Effect size measures were partial eta-squared (𝜂2p) in rm-ANOVA and Cohen’s d in post-hoc pairwise comparisons. Assuming a pooled standard deviation of 0.23 for BCVA, the study would require a sample size of 21 for each group (i.e. a total sample size of 42, assuming equal group sizes), to achieve a power of 80% and a level of significance of 5% (two-sided), for detecting a true difference in means between groups of 0.2 LogMAR at 6 months.

## Results

The analysis set comprised 42 subjects, 21 patients treated with ILM peeling (age: 67.7 ± 6.61) and 21 treated without ILM peeling (age: 66.3 ± 6.69). Sociodemographic, baseline and final clinical characteristics are extensively reported in Table 1. Macular hole closure was observed in all cases (100%) from month 1 (T1) and confirmed until the 6th months of follow-up (T3) in the “Peeling group”, whereas in the “No Peeling group” the 100% surgery success rate reported at the first month of follow-up (T1) was maintained until month 3 (T2), being the hole closure rate present in 19 subjects out of 21 (90%) at the six months follow-up (T3). Specifically, in the peeling group, after 1 month (T1), 10 out of 21 subjects (47,6%) had complete ELM/EZ recover, at the third month of treatment (T2) 16 out of 21 patients (76,2%) recovered at both ELM/EZ. At 6 months (T3) 18 out of 21 patients (85,71% ) had ELM/EZ recover. On the contrary, in the no peeling group a complete recover of ELM/EZ was observed in 6 out of 21 patients (28,57%) at one month (T1), 12 out of 21 patients (57,14%) at T2 and 15 out of 21 patients (71,43%) at T3. As for MPOD, when considering only the time factor statistically significant changes were reported within subjects over time (p < 0.001), however, no statistically significant differences between the two groups (peeling vs no peeling) were found in the post-hoc pairwise comparisons at all times. Similarly, when considering only time factor VDDCP significant variations were reported over time, (p = 0.010), however, without significant differences between the “peeling” and “ no peeling” group (*p* = 0.538). Besides, when evaluating VDSCP “peeling vs. no peeling” × “T0 vs. T1 vs. T2 vs T3” interaction factor, the rm-ANOVA was not significant (*p* = 0.835). The Mauchly sphericity test was significant: W = 0.159, 𝜒2 = 16.532, *p* < 0.001, thus indicating that the sphericity was violated. Post-hoc pairwise comparisons showed the absence of differences between groups (T0: pholm = 1.000; T1: pholm = 1.000; T2: pholm = 1.000 T3 pholm = 1.000). As regarding Visual Acuity, BCVA improved significantly in both peeling and no peeling groups (*p* < 0.001,) with no differences between the two groups (*p* = 0.841). The Mauchly sphericity test was not significant: W = 0.613, 𝜒2 = 4.411, *p* = 0.110, thus indicating that the sphericity was not violated. Specifically, according to the post-hoc pairwise comparisons analysis BCVA improves significantly only considering T0 and T3 time points, in both groups (peeling, T0 vs. T3: pholm = 0.002; no peeling, T0 vs. T3: pholm < 0.001) and no differences were found between peeling and no peeling groups (T0: pholm = 1.000; T1: pholm = 1.000; T2: pholm = 1.000; T3: pholm = 1.000). Finally, CMS significantly improved in both groups according to the rm-ANOVA model (*p* = 0.001,) and no differences appeared between the two interventions (*p* = 0.572,). The Mauchly sphericity test was not significant: W = 0.636, 𝜒2 = 4.066, *p* = 0.131, thus indicating that the sphericity was not violated.

**Table 1:** Demographic characteristics and baseline and final clinical characteristics

	Peeling *n* = 21		No peeling *n* = 21		*P* value
	*mean (SD)*		*mean (SD)*		
***Age***	67.7 (6.61)		66.3 (6.69)		
	*N (percentage*)		*N (percentage)*		
***Female***	13(61.90%)		10 (47.62%)		
***Type of Gas*** ***SF6***	21(100%)		21(100%)		
	*mean (SD)*		*mean (SD)*		
***Thinnest point ( µm)***	213 (37.8)		189 (53.4)		
***Axial lenght***	23.61 (0.68)		23.77 (1.95)		
	*mean (SD)*		*mean (SD)*		
	**T0**	**T3**	**T0**	**T3**	
***MPOD***	0.150 (0.059)	0.402 (0.061)	0.232 (0.163)	0.433 (0.143)	*0.390*
***VDDCP***	14.61 (4.375)	18.3 (2.08)	14.602 (2.188)	18.9 (2.011)	*0.835*
***VDSCP***	19.030 (7.029)	23.6 (3.41)	19.067 (5.184)	24.3 (2.20)	*0.538*
***BCVA*** ***(LogMAR)***	0.638 (0.225)	0.14 (0.200)	0.657 (0.216)	0.217 (0.200)	*0.841*
***CMS***	11.300 (3.453)	14.2 (1.308)	12.483 (1.974)	15.6 (1.82)	*0.572*

## Discussion

ILM removal in FTMH surgery has first been described by Eckardt et al. in 1997 [34]. Due to the fundamental structural role covered by Muller cells, this surgical maneuver is supposed to improve surgery success rate by reducing retinal rigidity, helping the vitreous cortex remnants removal on the ILM surface and preventing the fibrocellular proliferation that exploits the ILM as scaffold [35]. However, ILM peeling technique is not free of side effects. The occurrence of Dissociated Optic Nerve Fiber Layer appearance (DONFL) in the peeling area, the presence of Retinal Nerve Fiber Layer (RNFL) irregularities, Ganglion cell complex (GCL) thinning and retinal displacement are some reported anatomical consequences occurring after ILM peeling [22, 36–42]. More, functional defects as reduced retinal sensitivity, perifoveal microscotomas, alterations in visual acuity have yet been reported in literature [41]. The IVTS classification in 2013 by dividing FTMHs in small, medium and large, focuses on the presence or absence of vitreous attachment, thus enhancing the strong correlation between vitreous and FTMHs [11]. This correlation has already been hypothesized by Jondeeph et al. who reported VMT release by day 28 and hole closure by month 6 in case of small FTMHs associated with significant VMTs (29.9%, 41/137) [43].

In the present study, we have compared anatomical and functional changes occurring in eyes who underwent PPV with or without ILM peeling for sFTMH. Interestingly, although the overall majority of patients had a successful surgery with hole closure and improvement in visual function, a different closure rate was observed in the two groups, with surprising results at the sixth months follow-up. In fact, comparable results were reported until the third month of follow-up, with hole closure in all cases in both groups. However, 10% of patients in the no peeling group experienced the hole re-opening at the six months follow-up thus enhancing the ILM possible role in promoting this process. Similarly, in their multicenter randomized controlled trial Lois et al. found lack of differences in terms of functional outcomes when comparing ILM peeling and no peeling patients with idiopathic stage 2 or 3 FTMHs in a six months follow-up, concluding that, due to the higher anatomic closure and lower reoperation rate occurring in the ILM peeling group, ILM peeling can be indicated as treatment of choice in case of patients with idiopathic stage 2 to 3 FTMHs [44]. Besides, we found both EZ and ELM to be recovered in the majority of eyes in both groups during follow-ups. Specifically, the EZ/ELM recover rate was found to be higher in the “Peeling group” when compared to the “No Peeling group” from month one (T1) to the six months follow-up (T3). Similarly, Ruban et al. in their research found ELM and EZ recovery to be present at 3 months in 92% and 47% of eyes, respectively [1]. Buzzi et al. found a complete ELM reconstitution in the majority of cases, whereas residual EZ defects were reported [45]. Nevertheless, in the study they supposed this difference to be attributable to a baseline larger hole size, which prevents hole closure, whereas in our study this bias is overcome by the only enrollment of small macular holes. Tadayoni et al. had already investigated the relationship between macular hole size and potential benefits deriving from ILM peeling by observing that the aforementioned surgical maneuver seems not to be useful in case of FTMHs < 400 micron in diameter, since the lack of differences whether ILM was peeled off or not [46]. Furthermore, Hangai et al. hypothesized that interruption of ELM occurs when Muller cells cones are dragged away [47]. It has previously been demonstrated that ELM and EZ recovery are related to better visual acuity outcomes [48–50]. Thus, it has to be highlighted that also in our series significant improvements in BCVA have been reported in both groups, consistent with Bottoni et al. findings who likewise found increased BCVA in both groups, when comparing ILM peeling with ILM flap surgery [50].

Recent advancements in OCT-A have permitted a better comprehension of retinal diseases, including FTMHs. Rizzo et al. in their study evaluated vascular alterations occurring in eyes with FTMH finding the most frequent adjustments to be located in the DCP [51]. Michaelewska et al. analyzed OCTA images of eyes with FTMH before and after surgery reporting decreased blood flow at both. They hypothesized that the reduced VD around the hole in the deep retinal layers may be in some cases responsible for an incomplete functional recover especially occurring in long standing FTMHs [52]. In our study we found a VDSCP and VDDCP increase over time before and after surgery in both groups. Savastano et al. found VD in both SCP and DCP to be significantly related to BCVA both at baseline and six months after surgery, thus our results have to be highlighted, in order to underline the importance of VD in both DCP and SCP as a prognostic factor [53]. VD has previously been related to retinal sensitivity after macular hole surgery with superior inverted internal limiting membrane flap technique. Kunikata et al. in their retrospective study found lower retinal sensitivity recovery in the superior sector after surgery thus suggesting that internal limiting membrane peeling might affect the postoperative visual function [54]. We found a significant increased macular sensitivity in both groups after surgery, thus suggesting a good functional outcome in both groups. This is consistent with Romano et al. who found differences in macular sensitivity, although not statistically significant [55]. Due to the central role of Muller cells in the pathophysiological process of FTMH development and recover after surgery, a measurable index may be useful to study Muller cells redistribution occurring after repair, thus, the necessity of analyzing MPOD. In our study, MPOD globally changes across time in both groups. Romano et al. in their study found changes in MPOD in eyes with idiopathic Epiretinal Membrane (iERM) and FTMH treated with macular peeling with an only significant difference in FTMH patients [55]. They hypothesized that the anatomic closure of the hole leads to migration of cone photoreceptors and their axons, with consequent MP recovery in the foveal center and thus increased MPOD area and MPOD volume. This hypothesis is supported by Shiragami et al. as well [56]. Bottoni et al. found increased MPOD values after treatment of FTMH, with stable increased level over time [57]. It is well known that VMTs play an important role in FTMH genesis [58]. However, previous results have demonstrated that VMT release is not always accompanied by FTMH closure [7, 8]. As previously speculated, this condition may be attributable to the presence of not only the antero-posterior traction due to the evidence of VMTs, but also to the tangential forces originating from the presence of Epiretinal Membranes (ERMs) or persistent vitreous plaques, being responsible in causing or maintaining the hole [43, 59]. Besides, it is a common finding that eyes with small FTMHs frequently have an incomplete PVD. Thus, in this light we hypothesize that the only induction of intraoperative PVD, by removing main tractional forces, should be sufficient to obtain hole closure, as confirmed by our results. In this light, it is worth highlighting that by refraining from ILM peeling, unnecessary surgical stress on Muller cells may be avoided, thus permitting the foveal repair without any damage or interruption as demonstrated by our study where no functional differences were found between the two groups until six months of follow-ups. However, it must be assessed that the residual tangential traction due to the absence of ILM removal may not exclude the presence of a persistent tangential force that may be cause of hole re-opening observed in the no-peeling group at the sixth months follow-up, thus explaining the different anatomical outcome between the two groups. More, the incomplete ELM/EZ recover observed in the no peeling group may be considered as prognostic factor of a lower success rate in a long term. This study has some limitations: the sample size is relatively small, moreover, we performed cataract surgery is all the phakic patients enrolled in the study. Despite the distribution of cataract surgery was similar between the groups, the final BCVA could be biased by an improvement not related to the macular hole repair. Nevertheless, further studies on wider populations are welcome to validate and confirm our results.

## Conclusions

Concluding, this study shows the absence of significative anatomical and functional differences between the peeling or not the ILM in small FTMHs at 6 moths follow-up. Further studies are required to enhance the role of structural and functional parameters as prognostic factors and to better understand the dynamic of hole closure and what factors are involved in a successful surgery.

## Data Availability

Data are unavailable due to privacy restrictions.

## Abbreviations

FTMHs: Small Full Thickness Macular Holes
PPV: Pars Plana Vitrectomy
ILM: Internal Limiting Membrane
BCVA: Best Corrected Visual Acuity
EZ: Ellipsoid Zone
ELM: External Limiting Membrane
VD: Vessel Density
SCP: Superficial Capillary Plexus
DCP: Deep Capillary Plexus
VDSCP: Vessel Density in Superficial Capillary Plexus
VDDCP: Vessel Density in Deep Capillary Plexus
MPOD: Macular pigment Optical density
CMS: Central Macular Sensitivity
PVD: Posterior Vitreous Detachment
VMT: Vitreomacular Traction
VMA: Vitreomacular Adhesion
OCT: Optical Coherence Tomography
IVTS: International Vitreomacular Traction Study
ERG: Electroretinogram
LogMAR: Logarithm of the minimum angle of resolution
SD-OCT: Spectral Domain OCT
OCTA: OCT Angiography
MPOD: Macular Pigment Optical Density
MPS: Macular Pigment Screener
CMS: central macular sensitivity
EDI: Enhanced Depth Imaging
ROI: Region Of Interest
HFP: Heterochromatic Flicker Photometry
DONFL: Dissociated Optic Nerve Fiber Layer
RNFL: Retinal Nerve Fiber Layer
GCL: Ganglion cell complex
iERM: Idiopathic Epiretinal Membrane
ERMs: Epiretinal Membranes
